# Bladder mucinous adenocarcinoma as a diagnostic challenge: a case report

**DOI:** 10.11604/pamj.2022.42.221.35032

**Published:** 2022-07-20

**Authors:** Sofronievska Glavinov Maja, Kostadinova Kunovska Slavica, Abdiu Suad, Jovanovic Rubens

**Affiliations:** 1 Urology Department, University Surgical Clinic St. Naum Ohridski, Skopje, Republic of North Macedonia,; 2 Institute of Pathology, Medical Faculty, University Ss. Cyril and Methodius, Skopje, Republic of North Macedonia

**Keywords:** Mucinous adenocarcinoma, bladder, diagnosis, case report

## Abstract

Primary bladder adenocarcinoma (PBA), especially the mucinous subtype, is a rare cancer that represents less than 2% of all bladder malignancies. Overlapping histopathological and immunohistochemical (IHC) features of PBA with metastatic colonic adenocarcinomas (MCA) make the final diagnosis very hard. We presented a 75-year-old woman presenting with hematuria and severe anaemia in the last two weeks. The abdominal computed tomography scan showed a tumor-sized 2x2 cm right to the bladder dome. The patient underwent partial cystectomy without postoperative complication. The histopathologic and IHC showed mucinous adenocarcinoma and could not distinguish between the PBA from MCA. Investigations to exclude MCA revealed no other primary malignant site and suggested PBA. In conclusion, mucinous PBA requires ruling out any possibility of a metastatic lesion that could arise from other organs. Treatment should be considered an individual approach based on the tumour location and size, the patient´s age, general condition, and comorbidities.

## Introduction

Primary bladder adenocarcinoma (PBA), especially the mucinous subtype, is rare cancer that represents less than 2% of all bladder malignancies [1]. As a rare and devastating malignancy of the bladder, it accounts for an estimated 0.01% of all adult cancers [2]. The specificity of PBA is in the overlapping histologic and immunohistochemical (IHC) features with adenocarcinomas arising from other primary sites, especially colorectal and gynaecological malignancies [1,3].

The immunophenotype can also overlap with urachal neoplasms, especially in cases where the tumour is localized at the bladder dome [4]. Patients usually present with common symptoms such as recurrent urinary tract infections (UTI), irritative voiding, mucous-like discharge, and hematuria. Hematuria is the most common manifestation in more than 90% of patients, and surgery is the gold standard method for its treatment [5].

Few patients have been reported with mucinous PBA in the literature [6]. Here, we present a case of mucinous PBA in a 75-year-old woman. The clinical presentation, main treatments, and outcome of this case are discussed.

## Patient and observation

**Patient information:** a 75-year-old woman was referred from her primary care provider to the urology office due to massive hematuria with subsequent anaemia, dysuria, and fatigue. All symptoms started two weeks before hospital admission, but she had given information about intermittent episodes of painless hematuria in the previous six months. The patient mentioned a past medical history of three aortic bypasses, carotid endarterectomy, and chronic myocardiopathy, and she has been taking anticoagulant therapy for the past five years. The patient denied any weight loss, abdominal pain, or umbilical discharge. She did not report tobacco or alcohol, or drug abuse in her history. The patient family history of malignant diseases was negative. She is a retired secondary school teacher with healthy habits and was never exposed to harmful environmental factors. The patient was brought to the hospital by her spouse in a wheelchair, exhausted, and in poor general condition.

**Clinical findings:** the patient´s vital signs and physical examination pointed toward profound anaemia and hypovolemic shock (tachycardia with a pulse rate of 110 beats/min, tachypnea of 26 breathing /min, weak and filiform pulse, and pale skin). There was no palpable abdominal mass, but there was suprapubic tenderness and a visual globe.

**Figure 1:**
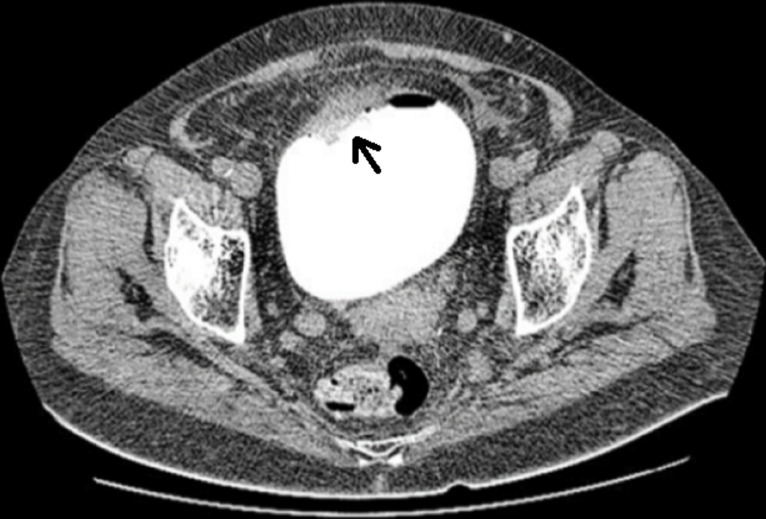
abdominal computed tomography scan with retrograde contrast showing the tumour mass (arrow)

**Figure 2:**
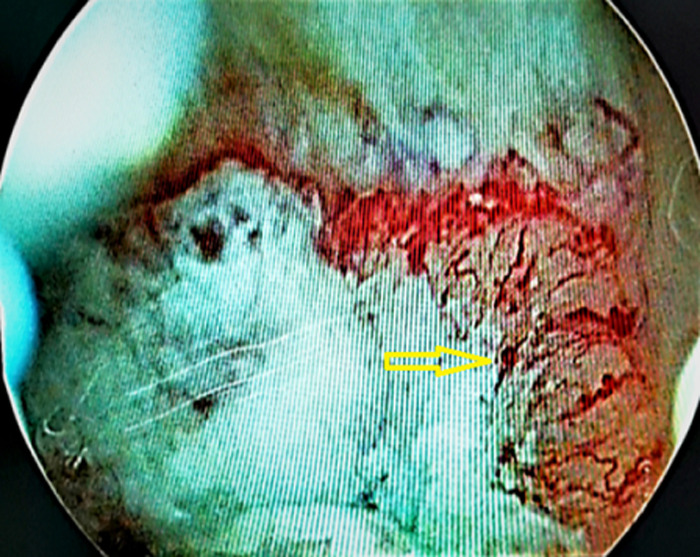
endoscopy view of the bladder mass (arrow)

**Figure 3:**
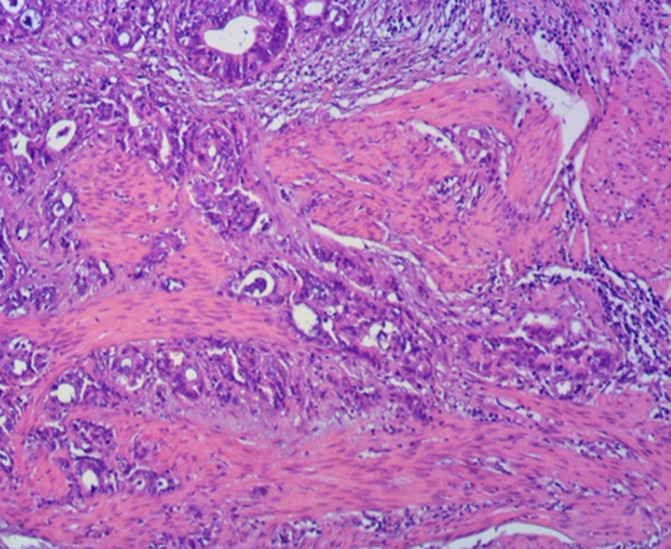
pathologic examination of the biopsy specimen showed abundant extracellular mucin with clusters of tumour cells floating in mucin lakes

**Figure 4:**
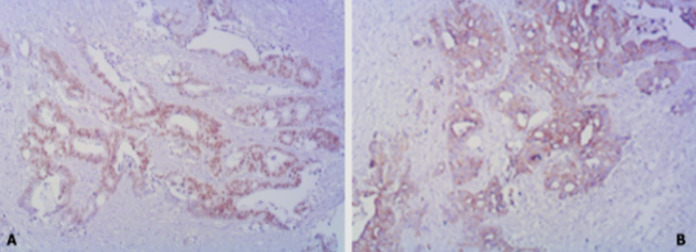
immunohistochemical staining (x40): A) positive CDX2, B) positive CK20

**Diagnostic assessment:** blood investigations showed severe anaemia (hemoglobin: 86 g/l and erythrocytes 2.44 x1012/l), urea: 15 mmol/l, and creatinine: 167 mmol/L. Blood coagulation status showed elevation of d-dimers: 4670 ng/ml and shortened thrombin time: 5.6 sec. Abdominal pelvic computed tomography (CT) scan with retrograde contrast injection showed intensively vascularized mass sized 2x2 cm, positioned right to the bladder dome (Figure 1). A 20 Fr triple silicone foley catheter was inserted in the bladder, and continuous irrigation with saline was applied. Then, the patient underwent explorative cystoscopy. First, massive cloths were evacuated, and exploration showed bladder mass arising from the dome. Consequently, a tumour biopsy was performed (Figure 2). Histopathological analysis of excised specimen showed an infiltrative malignant epithelial neoplasm composed of atypical glandular structures with nuclear and cytological atypia, and in some foci, the presence of mucin production (Figure 3). Immunohistochemical (IHC) analysis showed diffuse positivity for CDX2 and CK20 and partial positivity for CK7 (Figure 4). The primary diagnosis was bladder adenocarcinoma with high suspicion of intestinal origin. Investigations for the primary source of the tumour were performed such as tumour marker screening (CA 19-9, CA 125, alpha-fetoprotein, and CEA), colonoscopy, and pelvic magnetic resonance imaging (MRI) revealed no other primary malignant site or metastatic colonic adenocarcinomas (MCA).

**Therapeutic interventions:** after two months, the patient underwent partial cystectomy and bilateral lymphadenectomy.

**Figure 5:**
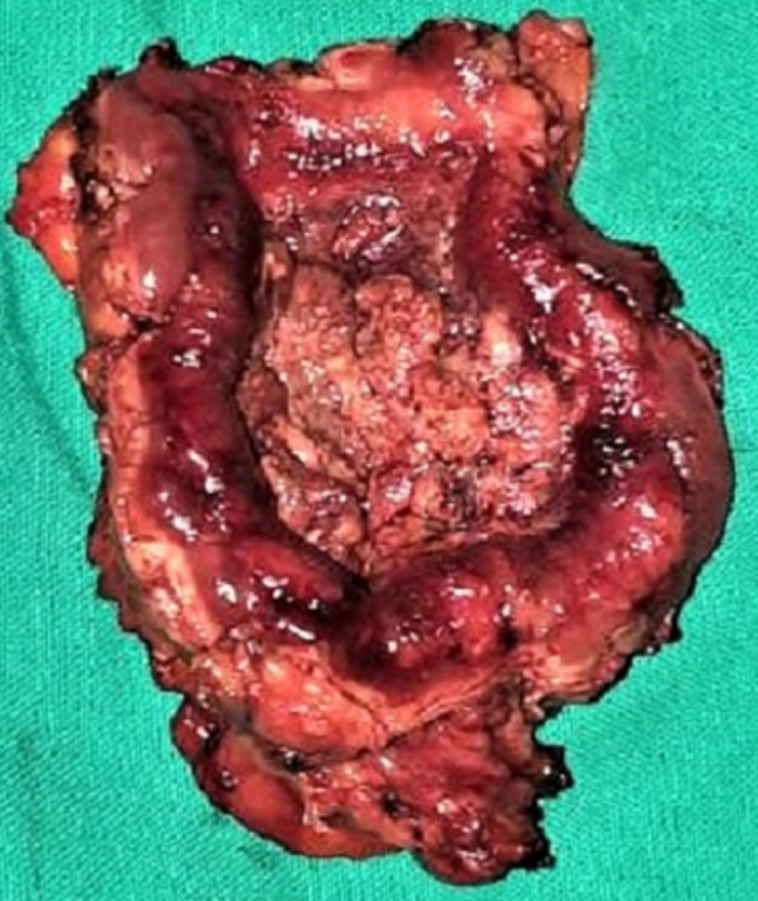
intraoperative photo showing the removed specimen

**Figure 6:**
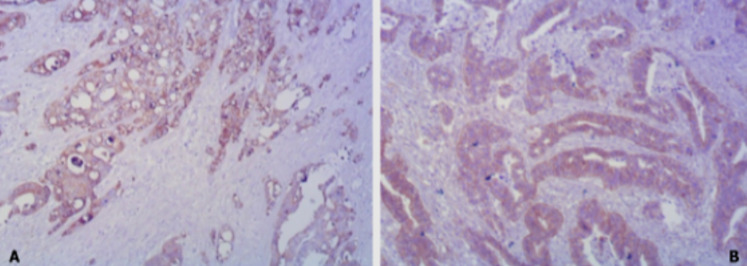
immunohistochemical staining (x40): A) positive CK7, B) positive beta-catenin

**Follow-up and outcome of interventions:** the patient´s postoperative recovery was satisfactory, and she was discharged after two weeks with an indwelling urinary catheter that was removed two weeks later. Histopathological examination of the resected specimen showed mucinous adenocarcinoma and IHC analysis could not exclude MCA (Figure 5). IHC showed CK20 and CDX2 expression, as in the biopsy specimen, the positivity of CK7 and Beta-catenin, and the focal expression of CA-125 (Figure 6). The tumour was negative for estrogen receptor, progesterone receptor, and Pax8. Therefore, the final diagnosis was primary mucinous bladder adenocarcinoma with pathological tumour-node-metastasis (pTNM) stage III, and pT3a, pN2, G2, NG2, L1, V0, and R0. Ambulatory one-shot catheter irrigation once weekly over four weeks with 20 ml Betaine/Polyhexanide solution diluted with saline 1: 2 was performed to avoid future UTIs. Antibiotic prophylaxis was prescribed with a nitrofurantoin night dose of 100 mg over a month. Since the tumour showed a poor response to chemotherapy and radiotherapy, regular cystoscopy follow-ups every three months during the first year and CT scan once a year for five years were recommended. After 2 years of follow-up, no signs of tumour recurrence or distant metastasis regarding cystoscopy and CT evaluations.

**Patient perspective:** I feel much better than ever. I went into this process with a high desire to feel better since my symptoms and fatigue began to impact everything I enjoy in life, but I was a little hesitant knowing my general condition. I thought it would be a difficult process, but it wasn´t once I established my lifestyle, diet, and supplement routines. I got my strength back and I can walk 6 kilometres per day again!”

**Informed consent:** written informed consent was obtained from the patient for participation in our study.

## Discussion

PBA of the bladder dome should be considered urachal cancer until proven otherwise [7]. However, when facing a mucinous PBA as a nonurachal bladder malignancy, it is necessary to exclude other possible primary locations since this type of tumour is extremely rare as a primary lesion, accounting for 0.5% to 2.0% of all malignant vesical tumours [1]. Mucinous PBA most commonly appears on the bladder dome, trigone, and lateral wall and is more common in schistosomiasis geographic regions and patients with a bladder of exstrophy [8]. Differentiating between mucinous PBA and urachal carcinoma is critical, but it can be difficult due to their similar presentations. Hematuria and dysuria are the primary clinical signs of PBA, as seen in our patient, whereas mucusuria is present in 90% of urachal carcinomas [8]. Given the rarity of PBA, relatively few studies have described its characteristics [7]. In our case, which factors contributed to the appearance of PBA remains unknown. As chronic irritation is one of the aetiology factors, we considered the possibility of underestimated chronic asymptomatic bacteriuria due to urinary catheter use after cardiovascular procedures as an intrinsic factor for bladder metaplasia [6].

Current clinical decisions on treating PBA are based on small case series and case reports [5]. Urachal carcinoma has a distinct natural history and clinical behaviour from nonurachal carcinoma. For patients with non-urachal bladder adenocarcinoma, radical cystectomy with pelvic lymph node dissection is the preferred treatment [5]. Additionally, there is no established evidence for the role of chemotherapy or adjuvant radiotherapy in treating PBA [9,10]. In our case, due to patient comorbidity and condition, the decision was made to perform en bloc partial cystectomy and bilateral lymphadenectomy. Hafizar *et al*. reported a similar procedure, which performed laparoscopic partial cystectomy in 60-year-old patients with urachal adenocarcinoma [11]. Morphologically, PBA causes a diagnostic dilemma for pathologists because it is difficult to differentiate from secondary involvement of the bladder by adenocarcinomas arising in adjacent organs, typically the colorectal, prostate, and female genital tract [12]. The overlapping IHC features of PBA, MCA, and urachal adenocarcinoma are well described in the literature. For example, Roy *et al*. reported that a strong nuclear with cytoplasmic-membranous -catenin staining was seen in 75% of MCA and only 16.7% of PBA (10% staining cells). Although abnormal nuclear E-cadherin staining was seen in both PBA and MCA. The authors concluded that CK-7, CK-20, Villin, and CDX-2 stains did not help distinguish between the two entities [8].

In another study, Wang *et al*. compared the IHC diagnosis of PBA and MCA [1^2^]. Positive beta-catenin was seen in 75% of the MCA versus 16.7% of PBA. In the same study, positive CK7, CK20, and CDX2 stains did not help distinguish the two entities [11]. In IHC study of our patient, CK7, CDX2, and beta-catenin were coexpressed, so a definitive distinction between PBA and adenocarcinoma of urachal origin could not be made. Besides these markers, CK20 was also positive, which showed difficulty in differentiation between the urachal origin of bladder adenocarcinoma and MCA. The final diagnosis was established by extensive diagnostic procedures aimed to exclude rather than confirm any gastrointestinal or gynaecological primary lesion. A similar result was reported by Fan and associations [13]. The prognosis of mucinous PBA depends mainly on its stage when diagnosed and treated. When the tumour is confined to the bladder, the survival rate is 75-100%; unfortunately, less than 30% of patients are diagnosed at an early stage. Because adenocarcinoma cells grow primarily through infiltration into the deep muscular layer and cystoscopy and B-mode ultrasound may not assess the extent of infiltration. As a result, most bladder mucinous adenocarcinoma patients are diagnosed at T2 or T3 stage, as seen in our patient [5,14].

## Conclusion

PBA is a very rare malignancy. In some cases, such as our case, when the IHC analysis is not conclusive in differentiating BPA from MCA, an extensive evaluation of the gastrointestinal and gynaecological tracts should be performed to establish a definitive diagnosis. Even though the recommended treatment for mucinous PBA is radical cystectomy, other less mutilate treatment options should be considered as an individual treatment approach that must include the tumour location and size, patient`s age, general condition, comorbidities, and the quality of life as the final goal of the treatment followed by meticulous patient follow-up.
